# Impact of radiofrequency power settings on acute mitral isthmus ablation success with concomitant vein of Marshall ethanol infusion

**DOI:** 10.1016/j.hroo.2026.04.003

**Published:** 2026-04-10

**Authors:** Antoine Da Costa, Cédric Yvorel, Vincent Roger, Cécile Romeyer, Rayan Mohammed, Antoine Carmaux, Kasra Azarnouch, Nathalie Grand, Marouane Bouhkris, Karim Benali

**Affiliations:** Department of Cardiology, Jean Monnet University, Saint-Etienne, France

**Keywords:** Persistent atrial fibrillation, Pulmonary vein isolation, Vein of Marshall, Ethanol infusion, Mitral isthmus, Mitral isthmus line, High-power short-duration, Power settings

## Abstract

**Background:**

Achieving acute bidirectional block across the mitral isthmus (MI) remains technically challenging owing to the complex architecture of the left atrial (LA) inferolateral wall and the presence of epicardial connections via the coronary sinus (CS) and vein of Marshall. Ethanol infusion into the vein of Marshall (VOM-EI) has been shown to facilitate acute MI line completion, but the influence of high-power short-duration (HPSD) radiofrequency (RF) settings in this context has not been fully evaluated.

**Objectives:**

This study aimed to assess the impact of 2 RF power strategies (50 W vs 35 W) on procedural efficacy and safety of MI ablation performed with adjunctive VOM-EI in an exploratory randomized study.

**Methods:**

71 patients planned for LA linear ablation for persistent atrial fibrillation were randomly assigned to receive either 50 W (HPSD; n = 37) or 35 W (conventional; n = 34). VOM-EI was the first step preceding LA RF. Procedural metrics, acute MI bidirectional block, and safety were analyzed.

**Results:**

Mitral RF delivery was performed with an ablation index target of 450–550. HPSD ablation significantly reduced the number of endocardial MI applications (11 ± 7.1 vs 18.8 ± 13; *P* < .002) and total MI applications (17.4 ± 16.4 vs 29.3 ± 23.3; *P* = .01) compared with 35 W, without increasing complications. First-pass endocardial MI block was achieved in 21 of 37 patients (56.8%) in the 50-W group and 11 of 34 (32.4%) in the 35-W group (absolute difference 24.4%; 95% confidence interval [CI], 2.1–45.3; *P* = .037). CS ablation was required less frequently in the 50-W group than in the 35-W group (43.2% vs 67.6%; absolute difference, −24.4%; 95% CI, −45.1 to −3.7; *P* = .038). CS applications when necessary did not differ significantly between 50-W and 35-W power (11 ± 10 vs 15.5 ± 13; *P* = .27). Acute reconnection rates were also comparable between groups (10% vs 16.6%; absolute difference, −6.6%; 95% CI, −23.2 to 10.0; *P* = .38). Acute MI block success rates were comparable between the 50-W and 35-W groups (97.3% vs 88.2%; absolute risk difference, 9.1%; 95% CI, −2.9 to 21.1; *P* = .13). Acute reconnection rates were also similar between groups (10% vs 16.6%; absolute risk difference −6.6%; 95% CI, −22.4 to 9.2; *P* = .38). Final MI block rates were similar between groups (97.3% vs 88.2%; absolute difference 9.1%; 95% CI, −5.4 to 23.6; *P* = .13).

**Conclusion:**

In this exploratory randomized study, a 50-W ablation strategy during MI ablation with adjunctive VOM-EI reduced the number of RF applications and increased first-pass block without increasing complications. These findings suggest that higher-power delivery may improve procedural efficiency during MI ablation. Larger studies are needed to determine whether these differences translate into improved long-term outcomes.

## Graphical abstract

**Figure:**
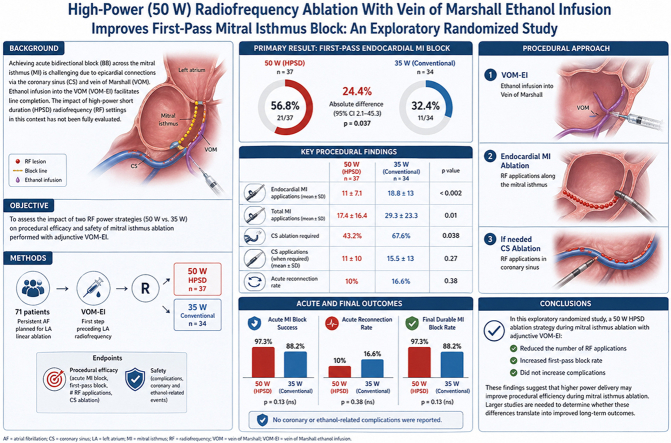

**Key Findings**

▪ This study evaluated the impact of radiofrequency power settings during mitral isthmus ablation performed within a workflow incorporating systematic vein of Marshall ethanol infusion.
▪ Both 35-W and 50-W strategies achieved high rates of acute bidirectional mitral isthmus block.
▪ The use of a 50-W high-power short-duration approach was feasible and improved procedural efficiency, with higher first-pass block rates and fewer additional applications, without increasing complications.
▪ These findings are limited to acute procedural outcomes and should be considered hypothesis generating.

## Introduction

After years of investigation, ablation strategies for persistent atrial fibrillation (PeAF) have evolved, with randomized studies supporting the role of adjunctive lesion sets, including mitral isthmus (MI) linear ablation and vein of Marshall ethanol infusion (VOM-EI), to improve rhythm outcomes.^1^, ^2^, ^3^, ^4^, ^5^, ^6^ Achieving reliable bidirectional block (BB) across the MI remains a key procedural endpoint when targeting perimitral circuits, and adjunctive techniques such as VOM-EI are increasingly incorporated into contemporary workflows.^7^, ^8^, ^9^ Achieving acute and durable BB across the MI remains 1 of the most technically challenging aspects of catheter ablation (CA) for treating left atrial (LA) arrhythmias.^7^, ^8^, ^9^, ^10^, ^11^, ^12^, ^13^ High-power short-duration (HPSD) radiofrequency ablation (RFA) has recently emerged as an alternative lesion delivery strategy, aiming to improve lesion quality and procedural efficiency compared with conventional radiofrequency (RF) settings.^14^, ^15^, ^16^, ^17^, ^18^, ^19^ By enhancing resistive heating and lesion contiguity, HPSD may facilitate linear ablation and improve acute MI block rates.^14^, ^15^, ^16^, ^17^, ^18^, ^19^, ^20^ However, the incremental benefit of using HPSD RF during MI ablation performed with adjunctive VOM-EI has not been well established, and comparative data between RF power strategies in this setting remain limited.

Therefore, this prospective randomized comparative cohort study aimed to assess the impact of RF power settings (50 W vs 35 W) on the acute efficacy and safety of MI ablation performed with adjunctive VOM-EI. The primary endpoint was procedural efficacy assessed by first-pass endocardial MI block, whereas secondary endpoints included procedural metrics, need for coronary sinus (CS) ablation, acute reconnection, and safety outcomes.

## Methods

### Study population

Consecutive patients diagnosed as having PeAF and scheduled for CA were prospectively enrolled at the University Hospital of Saint-Étienne, France, between September 2024 and September 2025. This study was conducted in accordance with the Declaration of Helsinki on ethical principles for medical research involving human subjects and was approved by the institutional review board of Saint Etienne University. All patients provided a written informed consent before participation. Consecutive patients were included if referred for first-procedure RFA for PeAF, regardless of its etiology, and selected for VOM-EI. Patients were randomly assigned in a 1:1 ratio to MI ablation using either 50-W or 35-W RF power. Randomization was performed before the procedure by an independent third party not involved in the ablation procedures, using a simple random draw. Treatment allocation was communicated to the operator at the beginning of the procedure after confirmation of eligibility. Because of the procedural nature of the intervention, operators were not blinded to group assignment. Patients with symptomatic, antiarrhythmic drug (AAD)–refractory PeAF were screened for eligibility for inclusion in this prospective cohort study. PeAF was defined as continuously sustained atrial fibrillation lasting beyond 7 days, including episodes resolved by pharmacologic or electrical cardioversion after 7 days.^21^

### Inclusion criteria

Patients were eligible for inclusion if they were aged 18 years and older, had symptomatic PeAF refractory to at least 1 AAD, and were undergoing a first-time CA procedure for PeAF. All included patients were in sinus rhythm at the time of the procedure, achieved by electrical cardioversion either the day before the intervention when performed under local anesthesia or on the same day when performed under general anesthesia.

### Exclusion criteria

The exclusion criteria were (1) paroxysmal atrial fibrillation, (2) long-standing PeAF lasting more than 3 years, (3) patients younger than 18 years, (4) intracardiac thrombus diagnosed by preprocedural transesophageal echocardiogram, (5) patients not providing informed consent, (6) absence of vein of Marshall (VOM) or failure to cannulate the VOM, and (7) contraindication to ethanol.

### Electrophysiological study and CA

All patients undergoing CA for PeAF were required to continue taking oral anticoagulants, including new oral anticoagulants or vitamin K antagonists, without interruption for at least 3 weeks before the procedure and until the day of the procedure. The electrophysiological procedure was performed with patients under conscious sedation or general anesthesia. Before LA access, VOM-EI was first performed as previously described, with minor modifications.^1^^,^^2^ In short, CS was cannulated with a large deflectable sheath (Agilis™, Abbot, St Paul, MN). CS opacification was performed with a 7.5F thermodilution Swan-Ganz balloon catheter (Edwards Way, Irvine, CA) in an anteroposterior view to identify VOM presence or absence. Then, a left internal mammary artery guide catheter was used for angiographic contrast injection and catheterization of the VOM ostium, which was then subselectively cannulated. An angioplasty guidewire (Sion® blue, Asahi Intecc USA, Santa Anna, CA) was advanced into the VOM, and a preloaded over-the-wire angioplasty balloon, 1.5, 2, or 2.5 mm in diameter (Sprinter™ OTW, Medtronic, Minneapolis, MN), was then advanced into the proximal VOM and inflated. VOM anatomy and occlusion were confirmed by a venogram. Afterward, if possible, a total dose of 9 mL of ethanol (100%) was slowly injected through the central balloon lumen at a rate of 3 mL per minute, and finally, the balloon was deflated. Then, transvenous access to the LA was achieved by transseptal puncture under fluoroscopic and/or echocardiographic guidance. A steerable decapolar catheter was placed into the CS for pacing and recording purposes. The steerable sheaths, diagnostic, and mapping catheters were continuously perfused with heparinized saline serum. After the transseptal puncture, a weight-based bolus dose of unfractionated heparin was administered, and additional boluses were given based on an activated clotting time of >300 seconds. Electroanatomic mapping was performed using a high-density diagnostic catheter (PENTARAY or OCTARAY, Biosense Webster, Irvine, CA) and a 3-dimensional mapping system (CARTO 3, Biosense Webster, Irvine, CA). A single transseptal puncture was performed, and catheter exchange was then performed for RFA using an open-irrigated 3.5-mm contact force–sensing RFA catheter (ThermoCool SmartTouch SF, Biosense Webster, Irvine, CA). Point-by-point ablation was performed in power-control mode (temperature limit 45°C; saline irrigation 8–20 mL/min). RF energy was delivered for 10–30 seconds with site-specific settings: 30 W within the CS, 40 W on the posterior wall, and up to 50 W on anterior sites. Automated lesion annotation was performed using the VisiTag module (catheter stability 2 mm for ≥4 seconds; minimum contact force 8 g for ≥70% of time). Lesion quality was guided by ablation index (AI) targets adapted to the anatomic region: posterior wall, AI 380; anterior wall, AI 450; and CS/epicardial applications, AI 380–400. A wide antral pulmonary vein isolation (PVI) approach was performed. 2 linear lesions were systematically created across the main LA isthmuses: roof line connecting the superior aspects of both PVI circles (40 W; AI target 380) and MI line from the left inferior pulmonary vein to the mitral annulus. Patients were randomized and assigned to RF delivery at 50 W (HPSD) or 35 W (conventional power) for MI ablation. For both power strategies, the target AI for endocardial MI applications was 450–550, with strict lesion contiguity (center-to-center interlesion distance <3 mm) and a target contact force of 8–30 g (minimum 8 g). The cavotricuspid isthmus line was not systematically performed. Roof line and MI BB were systematically assessed. The MI line was performed immediately after left-sided PVI. If bidirectional MI block was not achieved after VOM-EI and initial endocardial RFA, additional applications were delivered within the great cardiac vein and along the CS to target epicardial connections (30 W; AI 380–400) until block was obtained. Epicardial CS applications were delivered contiguously (<3 mm interlesion distance), with the catheter oriented toward the LA. All RF applications were delivered through a long steerable sheath (Agilis NxT, Abbott). Lesions not reaching the predefined AI target owing to instability were discarded and repeated to ensure a contiguous lesion set. After initial MI block, a 30-minute waiting period was observed to assess for acute reconnection. The procedural workflow was standardized as follows: VOM-EI → transseptal puncture → left PVI → MI ablation to BB → right PVI → roof line ablation. At the end of the procedure, PVI, roof line, and MI block were reassessed after a 30-minute waiting period (including time required for right-sided PVI and roof line ablation).

### Validation of procedural endpoints

MI BB was confirmed in both groups based on the following criteria: (1) a proximal-to-distal CS activation pattern during pacing from the LA appendage or the left lateral ridge and (2) activation from the LA lateral wall toward the ablation line during pacing from the distal CS or a longer stimulus–atrial interval at the LA appendage when pacing from the distal CS compared with pacing from the proximal CS (conventional differential pacing maneuvers). When necessary, MI block was also assessed and confirmed by differential pacing on both sides and along the line. PVI was confirmed by the absence of pulmonary vein electrograms or the presence of dissociated pulmonary vein electrograms unrelated to sinus rhythm. First-pass MI block was defined as the achievement of MI BB after the initial MI ablation, with an interlesion distance of 3 mm for linear lesions.

### Definition of the explanatory covariates

The clinical covariates used in this study were collected at baseline when patients were included. The CHADS-VA score, which includes congestive heart failure, hypertension, age of ≥75 years, diabetes mellitus, and previous stroke or transient ischemic attack, was also collected. Hypertension was defined as average blood pressure from 3 measures of >140 mm Hg systolic, >90 mm Hg diastolic, or blood pressure medication at inclusion. Diabetes mellitus and dyslipidemia were defined as antidiabetic and lipid disorder medication use, respectively. Obstructive sleep apnea was reported if the patient had a history of severe obstructive sleep apnea and/or chronic treatment with a continuous positive airway pressure machine. A preprocedural transthoracic echocardiography was performed to assess left ventricular ejection fraction; presence of dilated LA, defined as an LA area of >21 cm^2^; and aortic and mitral valve disease based on the presence of moderate or severe mitral and aortic regurgitation and/or stenosis.

### Postablation immediate management

Oral anticoagulation was continued after CA and thereafter, at the physician’s discretion, but was generally pursued in most cases except in cases of contraindication. The same AAD regimen was continued after the CA procedure until the first 2 months of clinical follow-up and was stopped thereafter. Before patient discharge, which occurred 24 hours after CA, rhythm assessment was conducted based on a 12-lead surface electrocardiogram.

### Statistical analysis

Categorical variables were expressed as percentages, whereas continuous variables were expressed as means ± standard deviations for parametric data and as medians (lower quartile to upper quartile) for non-normal data. Categorical data were analyzed using either a Fisher exact test or χ^2^ test, as appropriate. Unpaired data were analyzed using Student *t* test for parametric data and the Mann-Whitney *U* test for non-normal data. A 2-sided *P* value was determined where applicable, with a value of *P* ≤ .05 considered statistically significant. Effect sizes are reported as absolute differences between groups with corresponding 95% confidence intervals (CIs). All analyses were performed using StatView® 5.0 (StatView IV, Abacus Concept, Berkeley, CA) and XLSTAT software (Data Analysis and Statistical Solution for Microsoft Excel, Addinsoft, Paris, France, 2017).

## Results

### Patient population

71 patients who underwent the Marshall plan VOM-EI as first-line therapy were initially prospectively and consecutively included. Clinical and echocardiographic characteristics were as follows: mean age of 68 ± 8 years, 19.7% women, mean CHADS-VA score of 2.5 ± 1.6, mean left ventricular ejection fraction of 58% ± 12%, mean LA surface area of 25.3 ± 4.2 cm^2^, and mean indexed LA volume of 49.3 ± 17.7 mL/m^2^ (Table 1). Baseline clinical and procedural characteristics are presented according to randomization group in Table 1. The 50-W and 35-W groups were well balanced with no significant differences in baseline demographic, clinical, or echocardiographic variables (Table 1).

**Table 1:** Population characteristsics

Variable	Study population (n = 71)	50-W group (n = 37)	35-W group (n = 34)	*P* value
Age (y)	68 ± 8	67.4 ± 8.4	68.6 ± 6	.5
Gender (female)	14/71 (19.7)	5/37 (13.5)	10/34 (29.4)	.09
HTN	26/71 (36.6)	25/37 (67.6)	20/34 (58.8)	.6
DM	6/71 (8.4)	1/37 (2.7)	5/34 (14.7)	.07
CHA_2_DS_2_-VASc score	2.5 ± 1.6	2.3 ± 1.5	2.6 ± 1.7	.54
CAD	20/71 (28.2)	9/37 (24.3)	11/34 (32.4)	.45
BMI	27.5 ± 5	27.5 ± 5.7	27.4 ± 4.3	.9
Previous stroke	4/71 (5.6)	2/37 (5.4)	2/34 (5.9)	.9
PeAF duration (mo)	39 ± 30	35.1 ± 35	36.7 ± 32	.9
Left atrium surface, cm^2^	25.3 ± 4.2	25 ± 3.4	25.3 ± 4.9	.93
Left atrium volume, mL/m^2^	49.3 ± 17.7	49.3 ± 15.4	49 ± 20	.9
LVEF (%)	58 ± 12	57.4 ± 12.3	58.2 ± 11	.77

Data are presented as mean ± standard deviation, number (percentage), or median (interquartile range). BMI is the weight in kilograms divided by the square of height in meters.

BMI = body mass index; CAD = coronary artery disease; HTN = hypertension; DM = diabetes mellitus; LVEF = left ventricular ejection fraction; CHA_2_DS_2_-VASc = congestive heart failure, hypertension, age of ≥75 years (doubled), diabetes, stroke (doubled), vascular disease, age of 65–74 years, and sex category; PeAF = persistent atrial fibrillation.

### Procedure parameters

Procedural times (minutes) and fluoroscopy exposures (minutes) were as follows: total procedure (skin to skin) of 88.3 ± 29.4 and 9.9 ± 4.6 and VOM-EI of 12.6 ± 5.6 and 3.7 ± 2.7, respectively (Table 2). The total RFA duration application was 18 ± 11.5 minutes. The mean total number of RFA applications was 76.2 ± 38.4: 15 ± 11 for left PVI, 23.4 ± 15.7 for right PVI, 13 ± 9.7 for the roof line, and 23 ± 21 for the mitral line. Procedural characteristics are presented according to the randomization group in Table 2. The 35-W and 50-W groups were well balanced with no significant differences except for the number of right PVI applications (Table 2).

**Table 2:** Data on the RFA procedure

Procedure parameters	50-W group (n = 37)	35-W group (n = 34)	*P* value
Skin to skin (min)	86 ± 28	91 ± 31	.49
Total radiograph (min)	9.4 ± 4.5	10.4 ± 4.7	.37
VOM-EI (min)	12.7 ± 5.5	12.5 ± 5.7	.85
Total RFA time (min)	16.6 ± 11	19.7 ± 12	.27
Number of RF applications, left PVI (n)	16.2 ± 10	13.5 ± 13	.33
Number of RF applications, right PVI (n)	27.16	20 ± 15	.05
Number of RF applications, roof line (n)	13.3 ± 13.3	13 ± 9	.85

Values are expressed as mean ± standard deviation or number (percentage).

PVI = pulmonary vein isolation; RF = radiofrequency; RFA = radiofrequency ablation; VOM-EI = vein of Marshall ethanol infusion.

### Mitral line ablation

Mitral RF delivery was performed using an AI target of 450–550. Both groups (50 W vs 35 W) showed comparable values for mean impedance (108 ± 12 vs 106 ± 10 Ω; *P* = .44), overall impedance drop (9.0 ± 2.9 vs 8.6 ± 2.1 Ω; *P* = .40), and AI (504 ± 32 vs 492 ± 41; *P* = .20) (Table 3). High-power ablation significantly reduced the number of endocardial MI applications (11 ± 7.1 vs 18.8 ± 13; *P* < .002) and total MI applications (17.4 ± 16.4 vs 29.3 ± 23.3; *P* = .01) compared with 35-W power, without increasing complications (Table 3). First-pass endocardial MI block was achieved in 21 of 37 patients (56.8%) in the 50-W group and 11 of 34 (32.4%) in the 35-W group (absolute difference, 24.4%; 95% CI, 2.1–45.3; *P* = .037). CS ablation was required less frequently in the 50-W group than in the 35-W group (43.2% vs 67.6%; absolute difference, −24.4%; 95% CI, −45.1 to −3.7; *P* = .038). CS applications when necessary, did no differ significantly between 50-W and 35-W power (11 ± 10 vs 15.5 ± 13; *P* = .27). Acute reconnection rates were also comparable between groups (10% vs 16.6%; absolute difference, −6.6%; 95% CI, −23.2 to 10.0; *P* = .38). Acute MI block success rates were comparable between the 50-W and 35-W groups (97.3% vs 88.2%; absolute risk difference, 9.1%; 95% CI, −2.9 to 21.1; *P* = .13). Acute reconnection rates were also similar between groups (10% vs 16.6%; absolute risk difference, −6.6%; 95% CI, −22.4 to 9.2; *P* = .38). Final MI block rates were similar between groups (97.3% vs 88.2%; absolute difference, 9.1%; 95% CI, −5.4 to 23.6; *P* = .13). Both groups (50 W vs 35 W) were comparable with respect to LA volume (160 ± 43 vs 157 ± 42 mL; *P* = .76), MI length (37 ± 8 vs 39 ± 8 mm; *P* = .26), and the VOM-related low-voltage area on the voltage map (10 ± 8 vs 8 ± 7 cm^2^; *P* = .26) (Table 3). No coronary or ethanol-related complications were reported.

**Table 3:** Data on the MI ablation procedure

Procedure parameters	50 W (n = 37)	35 W (n = 34)	*P* value
Left atrial volume (mL)	160 ± 43	157 ± 42	.76
MI length (mm)	37 ± 8	39 ± 8	.26
VOM-related low-voltage area (cm^2^)	10 ± 8	8 ± 7	.2
Mean impedance (Ω)	108 ± 12	106 ± 10	.44
Overall impedance drop (Ω)	9.0 ± 2.9	8.6 ± 2.1	.4
Ablation index	504 ± 32	492 ± 41	.2
Total number of RF applications, MI (n)	17.4 ± 16.4	29.3 ± 23.3	.01∗
Number of RF applications, endocardial MI (n)	11 ± 7.1	18.8 ± 13	<.002∗
First-pass endocardial block	21/37 (56.8)	11/34 (32.4)	.037∗
CS application (yes/no) (%)	43.2	67.6	.038∗
Number of RF applications, CS (n)	11 ± 10	15.5 ± 13	.27
Distal CS pacing interval to LA (ms)	159 ± 25	174 ± 56	.13
LA pacing interval to distal CS (ms)	161 ± 29	174 ± 52	.21
Acute MI block success rates (%)	97.3	88.2	.13
Acute MI recovery rate (%)	10	16.6	.38
Final durable MI BB (%)	97	88	.1

Values are expressed as mean ± standard deviation or number (percentage).

BB = bidirectional block; CS = coronary sinus; LA = left atrium; MI = mitral isthmus; RF = radiofrequency; VOM = vein of Marshall.

∗ *P* < .05 significant.

### Complications

No steam pop was observed. Neither group experienced any serious adverse events during the procedures. Only 1 patient developed a moderate symptomatic pericardial effusion (9 mm) on day 1 after the procedure, which was successfully treated with corticosteroids and colchicine. The effusion resolved completely.

## Discussion

### Major findings

In this study of MI ablation performed within a workflow incorporating systematic VOM-EI, both 50-W and 35-W RF strategies achieved high rates of acute BB. A 50-W HPSD approach was feasible and improved procedural efficiency, with higher first-pass block and fewer additional applications, without safety concerns. Acute block remained stable during the 30-minute waiting period; however, given the modest sample size and absence of long-term remapping, these findings should be considered hypothesis generating.

### Impact of VOM-EI first on the MI block success

The VOM-EI strategy, initially conceptualized by Valderrábano and colleagues,^1^ has been shown to improve outcomes when added to PVI in PeAF, as demonstrated in the VENUS trial.^2^ Subsequent randomized studies and meta-analyses have supported the integration of VOM-EI into MI ablation strategies, reporting improved arrhythmia-free survival and higher rates of durable MI block.^1^, ^2^, ^3^, ^4^, ^5^, ^6^^,^^9^, ^10^, ^11^, ^12^, ^13^^,^^21^ More recent randomized studies have focused on the procedural role of VOM-EI.^7^^,^^8^ Gillis et al^7^ reported higher first-pass MI block rates when ethanol infusion was performed before RFA (94% vs 63%), suggesting that early VOM-EI facilitates MI ablation by creating a low-voltage substrate and improving visualization and cannulation of the VOM. Similarly, Zuo et al^8^ demonstrated improved acute MI block and reduced reconnection when VOM-EI was incorporated into the ablation workflow. In the present study, VOM-EI was systematically performed before MI ablation in both randomized groups, consistent with these procedural insights. Therefore, our results do not evaluate the incremental benefit of VOM-EI itself but rather compare RF power strategies within a standardized VOM-EI–guided workflow.

### HPSD ablation and MI

HPSD ablation has emerged as an alternative to conventional RF delivery, with several studies showing shorter procedure and RF times but similar long-term arrhythmia outcomes compared with conventional power strategies.^16^^,^^17^^,^^21^ Although HPSD may improve lesion contiguity and procedural efficiency, its specific role in MI ablation remains less well defined, particularly when adjunctive VOM-EI is incorporated. HPSD ablation primarily enhances resistive heating, leading to wider and more contiguous lesions rather than consistently deeper lesions. Lesion depth is generally influenced by multiple factors including contact force, catheter stability, tissue thickness, and duration of energy delivery.^14^^,^^21^, ^22^, ^23^, ^24^ Accordingly, HPSD does not necessarily produce deeper lesions than conventional power strategies but may improve lesion uniformity and reduce gaps along linear ablation lines. In the context of MI ablation combined with VOM-EI, these characteristics may facilitate completion of the endocardial lesion set rather than increase lesion depth per se. In our study, both RF strategies achieved high rates of acute MI block when VOM-EI was systematically performed first. The use of 50-W HPSD ablation was feasible and safe but was associated with greater procedural efficiency, including fewer additional applications to complete the MI line. However, given the modest sample size and the absence of a no-VOM-EI comparator, our results do not establish the superiority of HPSD over conventional power for MI ablation but rather support its feasibility within a VOM-EI–guided workflow. Larger studies are needed to determine whether higher-power strategies translate into improved long-term durability of MI block.

### Clinical implications

Our study found that the combination of VOM-EI with HPSD RFA at 50 W represents an effective strategy to facilitate durable MI block in patients with complex LA substrates such as the MI. By improving first-pass block and reducing the number of touch-up and ablation inside the CS, this approach may shorten procedure time, enhance lesion durability, and potentially reduce procedure-related complications. These findings support the use of optimized high-power settings in conjunction with adjunctive epicardial substrate modification by VOM-EI to improve the efficiency and reproducibility of mitral linear ablation.

### Limitations

This study has several limitations that should be acknowledged. First, although prospective, the study was conducted at a single center with a relatively limited sample size, which may reduce statistical power, particularly for detecting differences in less frequent safety outcomes and long-term efficacy endpoints. Given the sample size of the present study (37 vs 34 patients), the trial had sufficient precision to detect relatively large differences in procedural outcomes between groups (approximately 25%–30% absolute difference for binary endpoints), whereas smaller but potentially clinically relevant differences may not have been detected. Accordingly, the study may have been underpowered to detect modest differences between treatment strategies. Therefore, effect sizes with corresponding 95% CIs are reported to allow a more transparent interpretation of the magnitude and precision of the observed effects. Second, the analysis focused primarily on acute procedural endpoints, including first-pass acute MI block assessed during the index procedure, without systematic long-term rhythm follow-up; therefore, the impact of different power settings on late reconnection and clinical arrhythmia recurrence cannot be inferred. Third, all procedures were performed by experienced operators in a high-volume center, which may limit the generalizability of the results to centers with lower procedural volumes or less experience with VOM-EI. Fourth, ablation was performed using a predefined AI target, which may have mitigated differences between power settings and reduced the ability to isolate the independent effect of power delivery alone. In addition, epicardial substrate modification by VOM-EI was systematically performed before RFA, potentially attenuating differences in efficacy between groups and limiting extrapolation to MI ablation performed without VOM-EI. In addition, operators were not blinded to treatment allocation, which is inherent to procedural trials comparing ablation strategies and RF power settings. Although this lack of blinding could have influenced procedural behavior, objective endpoints such as achievement of acute bidirectional MI block and predefined AI targets were used to minimize potential bias. Finally, therefore, the results of this randomized study should be considered exploratory and hypothesis generating, and larger adequately powered studies are needed to confirm these observations.

## Conclusion

In this exploratory randomized study, a 50-W ablation strategy during MI ablation with adjunctive VOM-EI reduced the number of RF applications and increased first-pass block without increasing complications. These findings suggest that higher-power delivery may improve procedural efficiency during MI ablation. Larger studies are needed to determine whether these differences translate into improved long-term outcomes.

## Disclosures

The authors have no conflicts of interest to disclose.
